# Myelofibrosis: Treatment Options After Ruxolitinib Failure

**DOI:** 10.3390/curroncol32060339

**Published:** 2025-06-09

**Authors:** Ruth Stuckey, Adrián Segura Díaz, María Teresa Gómez-Casares

**Affiliations:** 1 Hematology Department, Hospital Universitario de Gran Canaria Dr. Negrín, 35019 Las Palmas de Gran Canaria, Spain; asegdia@gobiernodecanarias.org (A.S.D.); mgomcasf@gobiernodecanarias.org (M.T.G.-C.); 2 Medical Science Department, Universidad de Las Palmas de Gran Canaria, 35016 Las Palmas de Gran Canaria, Spain

**Keywords:** myelofibrosis, JAK inhibitors, ruxolitinib, resistant/refractory, novel therapies, personalized medicine, allogeneic hematopoietic stem cell transplantation

## Abstract

While allogeneic hematopoietic stem cell transplantation remains the only curative therapy for patients with myelofibrosis, its applicability is limited both by the high morbidity and mortality associated with the procedure and by the fact that only a minority of patients are eligible due to age or comorbidities. Ruxolitinib, a JAK1/JAK2 inhibitor, is the standard first-line therapy for intermediate- and high-risk MF, offering symptom relief and splenic volume reduction but lacking a clear survival benefit. Its use may be limited by hematologic toxicities, increased infection risk, and an inability to prevent disease progression. Ruxolitinib failure remains a significant clinical challenge, with resistance mechanisms not fully elucidated. The approval of other JAK inhibitors—fedratinib, pacritinib, and momelotinib—has expanded treatment options, particularly for patients with cytopenias or transfusion dependence. Moreover, many other targeted agents are in development in clinical trials, as monotherapy or in combination with ruxolitinib. This review provides an update on the use of JAK inhibitors and novel agents, with a focus on treatment options for ruxolitinib-resistant or refractory patients. As therapeutic strategies evolve, optimizing treatment sequencing and incorporating next-generation sequencing will be critical for improving patient outcomes.

## 1. Introduction

Myelofibrosis (MF), including primary and secondary MF (post-essential thrombocythemia, post-ET, and post-polycythemia vera, post-PV), is a highly heterogeneous disease in terms of its clinical presentation, general prognosis, and disease evolution [1]. In recent years, advances in our understanding of the molecular pathology of MF have permitted the more detailed genetic characterization of MF, leading to the refinement of diagnostic criteria and prognostic indices [2,3]. Moreover, such mechanistic studies have propagated the identification of novel molecular targets and spurred the development of novel pharmaceutical agents directed against them, which is the main scope of this review.

### 1.1. Disease Characteristics

The clinical manifestations of MF include splenomegaly, consequent to extramedullary hematopoiesis, cytopenias, and an array of potentially debilitating abdominal and constitutional symptoms that can significantly impact the patient’s quality of life [4,5].

The average age of presentation of PMF is 65 years, and the median estimated survival is 6.5 years [6], yet estimated survival is highly variable. The most common cause of death for patients with MF is transformation to acute leukemia, occurring in approximately 20% of patients [7], although many patients die from comorbidities, including thrombosis and cardiovascular complications, or as a consequence of cytopenias, including infection or hemorrhage [4,6]. Moreover, some 30% of patients with MF present with anemia at diagnosis, increasing to 47% who will develop marked anemia during follow-up [8].

### 1.2. First-Line Treatment Options

Considering the complex nature of MF and its highly variable phenotype, a well-informed individualized prognostic evaluation is necessary to determine the most appropriate treatment for patients with MF [1]. To date, allogeneic hematopoietic stem cell transplantation (allo-HSCT) is the only known potentially curative treatment for MF. Advances in conditioning regimes and donor selection have led to increasing numbers of HSCTs performed for MF in recent years [9]. For example, the number of HSCT per million of the population in Spain for 2022 was 76.7 pmp/year, up from 71.3 pmp/year in 2017, with 1.7% of transplants carried out in patients with myeloproliferative neoplasms [10]. Five-year overall survival rates now range from 40% to 70%, depending on risk profile, donor source, and transplant timing [10].

Nevertheless, the decision of whether to proceed with allo-HSCT is complex due to the high rates of transplant-associated morbidity and mortality—including transplant-related mortality of 20–30%, relapse rates of 15–25%, and chronic GVHD in up to 50% of long-term survivors [9]. Current guidelines recommend that PMF patients with intermediate 2- or high-risk MF (according to DIPSS, MIPSS 70, or MIPSS70+) or a low- or intermediate-risk MTSS score be considered candidates for transplant [3,11]. However, other groups only recommended allo-HSCT for patients with MF of intermediate-1 risk aged 65 years and under who have transfusion-dependent (or treatment-resistant) anemia, peripheral blasts > 2%, unfavorable karyotype and/or a triple-negative molecular profile with *ASXL1* mutation [12].

The decision on whether to transplant is complex and multifactorial and must also consider the patient’s general health and any prohibitive comorbidities (such as marked splenomegaly, thrombosis, or portal hypertension) [9,11,12]. Thus, in clinical practice, the majority of patients with MF are not candidates for allo-HSCT, and other treatment options need to be considered. For symptomatic patients for whom allo-HSCT is not an option, first-line options for the treatment of intermediate- or high-risk patients include ruxolitinib (approved by the FDA in November 2011 and by the European Medicines Agency in August 2012) for the treatment of splenomegaly and/or systemic symptoms. In contrast, for most patients with low-risk MF, the clinical approach is directed towards the management of symptoms, particularly to improve anemia and control the hyperproliferative manifestations of MF (splenomegaly, constitutional symptoms, leukocytosis, and thrombocytosis) predominantly with cytoreduction [1,3,4]. Thus, supportive therapies commonly used prior to the JAK inhibitor era—including androgens, erythropoiesis-stimulating agents, corticosteroids, and immunomodulatory drugs—continue to be relevant for selected patients, particularly those with symptomatic anemia or limited access to targeted agents.

## 2. Ruxolitinib Treatment

### 2.1. Advantages

The JAK1/JAK2 inhibitor ruxolitinib was developed as a novel targeted therapy following the demonstration of the essential role of the JAK/STAT pathway in the Philadelphia negative myeloproliferative neoplasms [13,14]. Until recently, ruxolitinib was the only approved pharmaceutical for MF (approved for the treatment of intermediate- or high-risk MF) due to clinical evidence supporting its efficacy in reducing splenomegaly and improving constitutive symptoms, in patients compared to placebo and best available therapy [15,16,17].

Importantly, results from the prospective, randomized, Phase III trials COMFORT-I and COMFORT-II showed that the clinical advantages do not seem to be affected by the patient’s age, type of MF (primary or secondary post-PV or post-TE), risk category, grade of splenomegaly, presence of cytopenias, or presence or absence of *JAK2* p.V617F point mutation [17,18]. However, although the FDA and EMA’s approval of ruxolitinib represents a major therapeutic advance in the field of myeloproliferative neoplasms (reviewed in [19]), evidence supporting the claim that ruxolitinib can prolong survival is relatively weak [20,21], and the treatment of MF patients with ruxolitinib does have various limitations.

### 2.2. Disadvantages

The principal disadvantage of ruxolitinib therapy is that it can cause the dose-dependent suppression of hematopoiesis as a result of non-selective JAK2 inhibition, with thrombocytopenia and anemia the most common adverse events. According to data from the COMFORT-II trial, 44.5% and 40.4% of patients were affected, respectively [15]. Both adverse effects tend to stabilize with dose adjustment but, in many cases, can lead to the suspension of ruxolitinib [22]. The splenic response to ruxolitinib has also been shown to be dose-dependent and has a median duration of approximately 3 years [23].

Furthermore, due to ruxolitinib’s immunosuppressive effects, treatment increases the risk of common bacterial and viral infections, including herpes zoster, pneumonia, bronchitis, and urinary tract infections, as well as the reactivation of tuberculosis and hepatitis B [24]. Indeed, the frequency of serious adverse event pneumonia among patients randomized to receive ruxolitinib was 15.5%, according to the 5-year update data from COMFORT-I [25].

Another important limitation associated with ruxolitinib treatment is its apparent inability to improve or prevent bone marrow fibrosis progression, an independent negative prognostic factor in MF. Short-term data from the COMFORT trials was not convincing, while more recent data from 68 patients enrolled in the INCB018424 trial (NCT00509899) suggests that 24 months of ruxolitinib treatment may delay or even reverse bone marrow fibrosis progression [26], although this observation remains to be confirmed in larger patient cohorts.

Finally, and perhaps most critically, treatment with ruxolitinib is unable to prevent progression to the blast phase. Five-year follow-up data from COMFORT-I found that 2.6% of ruxolitinib randomized patients (*n* = 5/155) progressed to AML, increasing to 3.6% in the ruxolitinib crossover group (*n* = 5/111), with a median time to AML diagnosis from a first ruxolitinib dose of 838 days and 376 days, respectively [25].

Indeed, approximately 50% of patients discontinue ruxolitinib therapy in the first 3 years due to serious adverse events or a loss of response associated with resistance, relapse, or blastic progression [27]. Notably, at the 5-year follow-up of patients included in COMFORT-I, only 27.7% (43/155) and 25.2% (28/111) of ruxolitinib randomized and crossed-over patients, respectively, were still on ruxolitinib treatment [25]. Treatment discontinuation was reported to be a result of adverse events for 16.8% and 9.9% of patients or disease progression in 14.8% and 19.8% of patients, respectively.

### 2.3. Failure

Failure of first-line ruxolitinib treatment is multifaceted but not well characterized, with a consensus definition of ruxolitinib failure an unmet need in current clinical practice. Pardanini and Tefferi examined several criteria that may help identify failure, which can manifest as disease progression, serious infection, severe bleeding, loss of spleen response, the development of acute cytopenias, and/or the continued need for blood transfusions [28]. A Delphi approach was used to reach a greater consensus on ruxolitinib failure in 2023, with specialists agreeing that failure could include no improvement in symptoms or spleen size, progressive disease, or ruxolitinib intolerance, following a maximally tolerated dose for ≥3 months [29].

Some studies have shown that ruxolitinib retreatment is safe and may even be beneficial in some ruxolitinib refractory or resistant patients. For example, a 2018 case series of patients with MF with loss of or inadequate response to ruxolitinib showed that retreatment with the drug resulted in a significant improvement in constitutional symptoms in 92% of patients and/or a reduction in spleen size in 69% of patients [30].

## 3. New Treatment Options

Prior to the approval of second-line treatment options for patients who were resistant, intolerant, or refractory to ruxolitinib, median survival after the discontinuation of ruxolitinib was estimated to be just 13.2 months [31]. However, treatment options for patients after ruxolitinib failure changed following the FDA’s approval of fedratinib in August 2019, pacritinib in February 2022, and momelotinib in September 2023. These approvals offer options for some previously difficult-to-treat patient subgroups, such as those with severe thrombocytopenia and transfusion-dependent patients. For example, momelotinib and pacritinib are recommended by the NCCN for the treatment of patients with platelets < 50 × 10^9^/L [32]. Momelotinib, recently approved, is mainly indicated in patients with anemia due to its additional mechanism acting on iron metabolism, while pacritinib is not yet available in Europe.

### 3.1. Fedratinib

Fedratinib, a Janus kinase 2 (JAK2), FMS-like tyrosine kinase 3 (FLT3), and bromodomain-containing protein 4 (BRD4) inhibitor, was approved by the FDA in August 2019 for the treatment of adult patients with intermediate-2 or high-risk primary or secondary MF. Unlike ruxolitinib, which inhibits both JAK1 and JAK2, fedratinib is selective for JAK2 while also targeting FLT3 and BRD4. This distinction suggests potential differences in efficacy and toxicity, although the two agents have not been directly compared in a randomized trial.

The JAKARTA and JAKARTA-2 trials provided the foundation for fedratinib’s approval [33,34]. In the phase 3 JAKARTA trial, treatment-naïve patients with MF experienced significant reductions in spleen volume and symptom burden compared to placebo. JAKARTA-2, which evaluated fedratinib in patients previously treated with ruxolitinib, demonstrated meaningful spleen volume reductions and symptom improvements, underscoring its efficacy as a second-line option. More recently, results from the FREEDOM2 trial (NCT03952039) provided further evidence of fedratinib’s efficacy in reducing spleen size and disease-related symptoms after ruxolitinib failure [35].

Beyond clinical trials, additional evidence has confirmed the benefits of fedratinib in MF patients after ruxolitinib discontinuation. A retrospective study involving 196 patients from Germany, Canada, and the United Kingdom highlighted its effectiveness in achieving spleen and symptom responses in the real world [36]. A review published in January 2025 summarizes the clinical trial data leading to fedratinib’s approval and real-world data on its use in patients resistant or refractory to ruxolitinib [37]. Importantly, the authors highlight the lack of real-world data on the efficacy of fedratinib in the first-line setting.

Fedratinib’s safety profile is distinct from ruxolitinib, with gastrointestinal adverse events such as nausea and diarrhea being more common. Strategies such as dose adjustments and prophylaxis can help mitigate toxicities and improve treatment adherence [35]. Additionally, the risk of Wernicke’s encephalopathy, a neurological disorder linked to thiamine deficiency, requires routine monitoring [38]. However, to date, no cases have been reported of withdrawal syndrome upon fedratinib discontinuation, a concern with abrupt ruxolitinib cessation [39,40].

One area of growing interest is the potential for sequential JAK inhibitor use. Some evidence suggests that reintroducing ruxolitinib following fedratinib discontinuation (due to intolerance or failure) may be feasible. A small study of 14 patients showed that some individuals could achieve symptom and spleen responses upon rechallenging with ruxolitinib after prior fedratinib treatment. Though response rates were lower than in first-line use and the cohort size was very small, these findings suggest that a sequencing approach is feasible and can offer patients some benefit [39].

### 3.2. Momelotinib

Momelotinib, a JAK1/JAK2 and ACVR1 inhibitor, was approved by the FDA in September 2023 for the treatment of patients with intermediate-2 or high-risk MF who also have anemia. Unlike other JAK inhibitors, momelotinib uniquely targets ACVR1 to suppress hepcidin expression, increasing iron availability for erythropoiesis to help improve anemia in MF patients [41]. This dual mechanism makes it a particularly valuable option for patients with transfusion dependence, addressing both disease burden and anemia-related complications.

Clinical trials, including SIMPLIFY-1, SIMPLIFY-2, and MOMENTUM, demonstrated momelotinib’s efficacy in reducing spleen volume, alleviating MF-related symptoms, and improving anemia in ruxolitinib-naive and previously treated patients [42,43,44]. Notably, in a phase 2 study, 41% of transfusion-dependent patients achieved transfusion independence for at least 12 weeks [45]. Additionally, its safety profile was favorable, with most adverse events—such as diarrhea and peripheral neuropathy—occurring in the first six months of treatment without cumulative toxicity [46].

Momelotinib offers a new therapeutic avenue for MF patients, yet its place in the treatment landscape remains under evaluation, particularly in relation to other JAK inhibitors like fedratinib and pacritinib. While momelotinib shows clear advantages in improving anemia and reducing transfusion dependence, spleen response rates appear to be somewhat lower compared to other JAK inhibitors [47], highlighting the need to individualize treatment based on symptom burden, cytopenias, and disease phenotype. Real-world data will continue to clarify its long-term benefits and optimal sequencing strategies in JAK inhibitor-experienced patients. In this regard, a recently published study of 154 MF patients treated with momelotinib (compassionate use) with symptom improvement in 92%, spleen reduction in 62%, and transfusion independence after 3 months for 48% and 58% of patients who were transfusion-dependent and transfusion-independent at baseline, respectively [47]. Together, these findings position momelotinib as a promising alternative to other JAK inhibitors, especially for patients with significant anemia. Furthermore, in the absence of the availability of pacritinib in Europe, momelotinib is the only JAK inhibitor with an indication for platelet counts ≥ 50 × 10^9^/L [3].

### 3.3. Pacritinib

Pacritinib, a selective JAK2, FLT3, and IRAK1 inhibitor, was approved by the FDA in 2022 for patients with intermediate- or high-risk myelofibrosis (MF) with severe thrombocytopenia (platelet counts < 50 × 10^9^/L), a subpopulation of patients excluded from previous clinical trials with ruxolitinib and fedratinib. Pacritinib has the advantage of having minimal activity against JAK1 at doses that inhibit JAK2 [48], and so is not myelosuppressive, unlike ruxolitinib, fedratinib, and momelotinib. Moreover, unlike other JAK inhibitors, pacritinib also has potential anti-inflammatory effects through IRAK1 inhibition.

Initial concerns over bleeding and cardiovascular events led to a clinical hold of the PERSIST-1 and -2 studies in 2016, but further studies, including PAC203, demonstrated improved safety with lower doses [49,50,51]. Approval was granted when pooled analyses of data from clinical trials, particularly PERSIST-2, showed pacritinib’s efficacy in reducing spleen volume symptom burden for patients with anemia or thrombocytopenia at baseline, as well as previously ruxolitinib-treated patients [50]. Significantly, these clinical improvements in pacritinib treatment were shown to positively impact the survival of patients [52]. Ongoing trials like PACIFICA (NCT03165734) will continue to clarify pacritinib’s clinical benefits and safety profile, particularly in patients with significant thrombocytopenia.

### 3.4. Other Novel Agents

Ruxolitinib combination therapies may be an appropriate alternative in some patients. Moreover, several other investigational JAK1/2 inhibitors are in the development pipeline, such as the JAK2 inhibitors NS-018 and gandotinib and the JAK1 inhibitor itacitinib.

The European Commission granted Orphan Drug Designation to NS-018 (ilginatinib) in August 2023. This oral, selective JAK2 inhibitor is currently being evaluated in a phase 2b randomized controlled trial versus the best available therapy for MF patients with severe thrombocytopenia (NCT04854096).

Gandotinib (LY2784544), an oral JAK2 inhibitor, has shown dose-dependent selectivity for the *JAK2* p.V617F mutation as well as other *JAK2* mutations. A phase 2 trial (NCT01594723) demonstrated symptom score improvements after 12 months. Although spleen reductions were reported, they were not as significant as with ruxolitinib [53]. However, gandotinib exhibited a favorable safety profile, with lower myelotoxicity and fewer infections compared to ruxolitinib and other JAK inhibitors.

Jaktinib has also emerged as a promising option for MF patients who are refractory to or have relapsed after ruxolitinib treatment. A phase 2 trial evaluating jaktinib 100 mg BID reported a spleen volume response (SVR) ≥ 35% rate of 32.4% at week 24, with 46.4% of patients achieving a ≥50% decrease in total symptom score (TSS). The most common grade ≥ 3 treatment-emergent adverse events were thrombocytopenia and anemia (each 32.4%) [54]. Additionally, the ZGJAK002 phase 2 trial assessed jaktinib at different dosing regimens in JAK inhibitor-naïve patients, demonstrating efficacy in spleen reduction, symptom relief, and anemia improvement across various patient cohorts [55].

Another promising agent with a novel mechanism of action is INCB160058, which exhibits a specific affinity for the *JAK2* p.V617F mutation. By binding to the mutant protein, it prevents ligand-independent thrombopoietin receptor dimerization [56], a key driver of disease pathogenesis. This targeted approach aims to selectively inhibit mutant clones while preserving endogenous JAK2 function in cytokine signaling. Data on its efficacy are limited to date, but its potential is being explored in an ongoing Phase 1 clinical trial (NCT06313593).

Many other novel agents are currently being investigated (many in combination with ruxolitinib) for the second-line treatment of MF patients (for a recent comprehensive review, see [57]). For example, at our center alone in the last year, five clinical trials have evaluated or are evaluating therapies for patients who are ruxolitinib resistant/refractory, including navtemadlin in monotherapy (NCT03662126) or in combination (NCT06479135), INCB057643 in monotherapy (NCT04279847), navitoclax in combination (NCT04468984), and imetelstat in monotherapy (NCT04576156), while four others are evaluating first-line treatment of ruxolitinib-naive MF patients, including navitoclax in combination (NCT04472598), pelabresib in combination (NCT04603495), selinexor in monotherapy (NCT05980806) or combination (NCT04562389; Table 1).

Moreover, other ongoing trials with drugs such as luspatercept (NCT04717414) and sotatercept (NCT01712308) have the aim to improve anemia and reduce the requirement of red blood cell transfusions of MF patients on ruxolitinib treatment. Indeed, recent results from the ACE-536-MF-001 trial showed that both transfusion-dependent and non-transfusion-dependent patients benefited from improved anemia and a reduction in TSS with luspatercept [58].

## 4. Discussion

Patients diagnosed with pre-fibrotic/early PMF may not be eligible for or benefit from treatment with JAK inhibitors [59,60,61]. Furthermore, patients with low or intermediate-1 risk without splenomegaly may not be well suited to treatment with JAK inhibitors due to potential side effects, including infections, cytopenias (neutropenia, thrombocytopenia, and anemia), gastrointestinal issues, and potential concerns of non-melanoma skin cancer [62]. Thus, the treatment options for these patients with early or lower-risk PMF are limited and often involve an unsatisfying “watch-and-wait” management approach.

Nevertheless, advancements in pegylated interferons have led to renewed interest in their use as a treatment option for lower-risk MF. Ropeginterferon alfa-2b (ropeg) is a new-generation pegylated interferon-based therapy with favorable pharmacokinetics and safety profiles, requiring less frequent injections than prior formulations. The phase 1/2 RUXOPEG study (NCT02742324) evaluating ruxolitinib with pegylated interferon-alpha 2a (pegIFNa-2a) in JAK inhibitor-naïve MF patients reported a 50% reduction in spleen length in 70% of participants [63]. Additionally, ropeginterferon alfa-2b, next-generation pegylated interferon, is being investigated in an ongoing randomized phase 3 trial for early/lower-risk (pre-fibrotic or low- or intermediate-1 risk PMF patients, according to DIPSS-plus) [64].

With the availability of multiple JAK inhibitors approved for myelofibrosis treatment (four by the FDA and two by EMA) and many others in the pipeline, clinicians must carefully consider patient-specific factors, including platelet count, transfusion dependence, and symptom burden, to determine the most suitable therapy (Table 2) [65]. The choice of JAK inhibitor is increasingly guided by platelet thresholds, with different agents recommended for varying degrees of thrombocytopenia. For example, ruxolitinib, fedratinib, and momelotinib are viable options for patients with platelet counts ≥ 50 × 10^9^/L, while momelotinib and pacritinib are recommended for those with lower platelet counts. A comparative meta-analysis highlighted variations in JAK inhibitor effects on blood cell counts; momelotinib carries a lower anemia risk, whereas pacritinib is more likely to cause thrombocytopenia. While pacritinib was less effective than ruxolitinib in first-line treatment, it demonstrated promise in second-line therapy after ruxolitinib exposure, reinforcing the need for individualized treatment selection [66].

## 5. Conclusions

The approvals of fedratinib, momelotinib, and pacritinib, along with the increasing number of molecular targets under active investigation, are transforming MF treatment. While ruxolitinib remains a cornerstone, its limitations, particularly in advanced disease stages with cytopenias, necessitate alternative strategies. The development of second-generation JAK2 inhibitors with reduced myelosuppression may extend treatment benefits to a broader patient population, offering new hope and personalized therapeutic strategies for patients with ruxolitinib-resistant or refractory disease.

## 6. Future Directions

Ongoing research continues to refine treatment sequencing, with a focus on improving patient outcomes and optimizing treatment decisions while minimizing adverse effects. Moreover, until recently, primary endpoints of clinical trials in MF have mostly included spleen and symptom response. Given the large number of novel agents and ruxolitinib combination approaches, new trials should also aim at improving event-free or overall survival. Longer follow-up of these new treatment options will be required to determine the effects on overall survival.

A greater understanding of the molecular pathogenesis of MF has informed the development of novel therapeutic agents. Nevertheless, to date, the mechanisms of failure, including primary resistance, i.e., no clinical response within 28 days of commencing ruxolitinib treatment, remain to be elucidated. Possible mechanisms that may contribute to resistance to ruxolitinib include adaptive resistance via heterodimer formation between activated JAK2 and other JAK-family kinases, including JAK1 or TYK2 [67], or the activation of alternative signaling pathways, such as PI3K/AKT/mTOR and RAS/MAPK (reviewed in [68]). Interestingly, no specific acquired *JAK2* mutations in patients associated with ruxolitinib resistance have been identified to date. However, molecular studies suggest that the presence of one or more high-risk mutations (such as *ASXL1*, *EZH2*, *IDH1/IDH2*, and/or *SRSF2*) can significantly reduce the time to ruxolitinib treatment discontinuation as well as negatively impact on leukemic transformation and overall survival [69,70]. Furthermore, MF patients with three or more mutations at baseline were more prone to ruxolitinib failure. Specifically, patients with multiple mutations were less likely to achieve a spleen response and had a shorter time to ruxolitinib discontinuation [70]. Thus, clonal evolution with the acquisition of additional mutations may contribute to resistance to ruxolitinib.

Given the expanding number of treatment options available for MF patients, early predictors of inferior survival post-ruxolitinib are crucial for timely intervention. The RR6 prognostic model, based on data from the RUXOREL-MF study (NCT03959371), identified impaired survival predictors such as <30% spleen length reduction at months 3 and 6 and red blood cell transfusion requirement as predictors of survival after six months of ruxolitinib treatment [71]. These insights support the need for prompt treatment shifts—more so now than ever as other options are available—rather than prolonged ruxolitinib use beyond first-line failure. Furthermore, the molecular characterization of patients with MF with methods such as next-generation sequencing will play an increasingly important role in MF management in the future, and treatment decisions for MF patients should be supported by predictive algorithms, in particular after ruxolitinib failure [72].

## Figures and Tables

**Table 1:** Novel agents were investigated in ongoing clinical trials at our center, in monotherapy or combination with ruxolitinib for the first-line treatment of myelofibrosis patients or for the second-line treatment of patients who are resistant or refractory to ruxolitinib.

Drug	First- or Second-Line	Monotherapy or Combination	Comparative Arm	Phase	Clinical Trial Code	Primary Endpoint
Navtemadlin	Second	Monotherapy	BAT	3	NCT03662126	SVR of ≥35% at Week 24
Navtemadlin	Second	Combination	Placebo plus Ruxolitinib	3	NCT06479135	SVR of ≥35% and TSS reduction ≥ 50% at Week 24
INCB057643	Second	Monotherapy	-	1	NCT04279847	Safety and tolerability
Navitoclax	First	Combination	Placebo	3	NCT03222609	SVR of ≥35% at Week 24
Navitoclax	Second	Combination	BAT	3	NCT04468984	SVR of ≥35% at Week 24
Imetelstat	Second	Monotherapy	BAT	3	NCT04576156	Overall survival
Pelabresib	First	Combination	Placebo plus ruxolitinib	3	NCT04603495	SVR of ≥35% at Week 24
Selinexor	First	Monotherapy	-	2	NCT05980806	SVR of ≥35% at Week 24
Selinexor	First	Combination	Placebo plus ruxolitinib	3	NCT04562389	SVR of ≥35% and TSS reduction ≥ 50% at Week 24
Luspatercept	Second	Combination	Placebo	3	NCT04717414	RBCT-free > consecutive 12-week period between randomization and Week 24

BAT: best available therapy; RBCT: red blood cell transfusion; SVR: spleen volume reduction; TSS: total symptom score.

**Table 2:** Current JAK-inhibitor treatment options for myelofibrosis based on the clinical scenario.

Scenario	Recommended Treatment	Key Benefits	Limitations
Front-line therapy for MF	Ruxolitinib (JAK1/JAK2 inhibitor) OR Fedratinib (JAK2/FLT3/BRD4 inhibitor) OR Momelotinib (JAK1/JAK2/ACVR1 inhibitor)	Ruxolitinib: Reduces spleen size and symptom burden. Fedratinib: Alternative first-line option; selective JAK2 inhibitor. Momelotinib: Ideal for patients with moderate to severe anemia	Ruxolitinib: Myelosuppressive; withdrawal syndrome risk Fedratinib: Gastrointestinal toxicity; Wernicke’s encephalopathy risk Momelotinib: Lower spleen response rates compared to other JAK inhibitors
Ruxolitinib failure	Fedratinib (JAK2/FLT3/BRD4 inhibitor) OR Momelotinib (JAK1/JAK2/ACVR1 inhibitor)	Fedratinib: Effective second-line option; reduces spleen volume. Momelotinib: Preferred for anemia/transfusion dependence after ruxolitinib failure	Fedratinib: Gastrointestinal toxicity; Wernicke’s encephalopathy risk. Momelotinib: Lower spleen response rates vs. other JAK inhibitors
Severe thrombocytopenia (platelets < 50 × 10^9^/L)	Pacritinib (JAK2/FLT3/IRAK1 inhibitor)	Minimal myelosuppression; suitable for patients with low platelet counts	Gastrointestinal toxicity; cardiac concerns
Transfusion dependence/significant anemia	Momelotinib (JAK1/JAK2/ACVR1 inhibitor)	Improves anemia via ACVR1 inhibition; reduces transfusion dependence	Lower spleen response rates compared to other JAK inhibitors
